# Epidemiology and Outcomes of Hospitalized Inflammatory Bowel Disease Patients with Asthma in the United States

**DOI:** 10.7759/cureus.6127

**Published:** 2019-11-11

**Authors:** Gebran Khneizer, Ahmad Al-Taee, Kahee Mohammed, Christine Hachem

**Affiliations:** 1 Internal Medicine, Indiana University Hospital, Indianapolis, USA; 2 Internal Medicine, Saint Louis University, St. Louis, USA; 3 John T. Milliken Department of Medicine, Washington University School of Medicine, St. Louis, USA; 4 Gastroenterology, Saint Louis University Hospital, St. Louis, USA

**Keywords:** inflammatory bowel disease (ibd), asthma, hospitalization, socio-demographic and clinical characteristics, length of stay (los), in-hospital mortality

## Abstract

Introduction

Research on the epidemiology and outcomes of hospitalized inflammatory bowel disease (IBD) patients with a history of asthma in the United States (US) is limited. This study aimed at identifying the sociodemographic and clinical characteristics of hospitalized IBD patients with a diagnosis of asthma. We also examined the association between an asthma diagnosis and the length of stay (LOS) and in-hospital mortality among hospitalized IBD patients.

Method

Using the National Inpatient Sample (NIS) for the years 2008-2013 and the ninth edition of the International Classification of Diseases codes, we identified adult hospitalized patients with IBD (N = 370,636) and used weighted multilevel hierarchical logistic regression models.

Results

The overall prevalence of asthma in our cohort of hospitalized IBD patients was 8%. Hospitalized IBD patients with asthma were more likely to be female, <45 years old, have Crohn's disease, and a higher Elixhauser comorbidity index (ECI). IBD patients with ECI of 3 or more had higher odds of having a prior diagnosis of asthma compared to those with no comorbidities (OR 63.33, 95% CI: 54.51-73.58). Having a prior diagnosis of asthma among hospitalized IBD patients was associated with lower odds of prolonged hospital stay and in-hospital mortality (OR 0.72, 95% CI: 0.69-0.74; OR 0.49, 95% CI: 0.43- 0.56, respectively). Patients with both IBD and asthma are more likely to seek medical care with earlier and aggressive treatment modalities, which may explain the lower in-hospital mortality in this group.

Conclusion

Lower in-hospital mortality and geographic variation are notable in the outcomes of IBD patients with asthma. Future prospective studies are necessary to improve our understanding of the management and interplay of IBD patients with asthma.

## Introduction

Inflammatory bowel disease (IBD) and asthma are both immune-mediated conditions that may arise from complex interactions between genetic predisposition and environmental factors. Both diseases have been shown to share a number of susceptibility genes. Moreover, the role of hygiene hypothesis has been proposed for both conditions. This may potentially explain the increasing incidence of both conditions in the 20th century with improvements in sanitary conditions. Several observational studies and systematic reviews have previously described the association between asthma and IBD including both Crohn's disease and ulcerative colitis. However, it is still unclear if one disease may influence the risk of developing another disease.

Data on the epidemiology and outcomes of hospitalized IBD patients with a history of asthma in the US is lacking. Previous studies have focused on non-US outpatient samples [1-2]. This study aimed to explore the trends in epidemiology and outcomes of hospitalized IBD patients with a history of asthma. To our knowledge, this is the first study to utilize an inpatient US cohort.

## Materials and methods

This study aimed at identifying the sociodemographic and clinical characteristics of hospitalized IBD patients with a concomitant diagnosis of asthma. Additionally, we examined the association between an asthma diagnosis and the length of stay and in-hospital mortality among hospitalized IBD patients. We utilized the National Inpatient Sample (NIS) for the years 2008-2013. The NIS database is the largest publicly available all‐payer inpatient care database in the US, containing data on more than seven million hospital stays each year. Using the ninth edition of the International Classification of Diseases codes, we identified adult hospitalized patients with IBD (N = 370,636). Weighted multilevel hierarchical logistic regression models were used to examine the sociodemographic and clinical characteristics of asthmatic IBD patients (n = 29,720) and evaluate the impact of a prior diagnosis of asthma on the length of hospital stay and in-hospital mortality. Hospital length of stay (LOS) was dichotomized into a prolonged hospital stay (yes/no) if the LOS was ≥75th percentile based on the presence or absence of a significant operating room procedure related to the same admission for each patient.

## Results

The overall prevalence of asthma in our cohort of hospitalized IBD patients was 8%, a statistically significant increase from 7.6% in 2008 to 9% in 2013 (*p *< 0.05). Hospitalized IBD patients with asthma were more likely to be female, <45 years old, have Crohn's disease, and a higher Elixhauser comorbidity index (ECI; Table 1). 

**Table 1:** Descriptive data of the sample of IBD patients according to the diagnosis of asthma yr., year; UC, ulcerative colitis; CD, Crohn's disease; IBD, inflammatory bowel disease

Characteristics	All IBD	Without Asthma	With Asthma	P value
Number of patients, N (Weighted %)	370,636	340,916 (92)	29,720 (8)	-
Sex (%)				
Male	30.5	40.5	2.45	<0.001
Female	69.5	51.53	5.57	
Age (yr.) (%)				
<45	37.4	34.3	3.1	<0.001
45-64	33.1	30.1	3	
≥65	29.4	27.5	1.9	
Race/ethnicity (%)				
White	80.2	74.1	6.1	<0.001
Black	10.38	9.3	1.1	
Hispanic	5.52	5	0.5	
Other	3.9	3.6	0.3	
Region (%)				
Northeast	22.5	20.5	2	<0.0001
Midwest	25.6	23.4	2.2	
West	16.4	15.1	1.4	
South	35.5	33	2.5	
IBD Subtype (%)				
UC	36.6	33.9	2.7	<0.0001
CD	36.4	58.1	5.4	
Elixhauser comorbidity index (%)				
C0	18.5	18.5	0.06	<0.0001
C1	22.8	21.5	1.3	
C2	21.1	19.3	1.74	
C3	37.6	32.7	4.9	

IBD patients belonging to the African-American race or Hispanic ethnicity had higher odds of having a diagnosis of asthma when compared to Caucasian patients (OR 1.24, 95% CI: 1.19-1.29 and OR 1.18, 95% CI: 1.11-1.25, respectively). When compared to the age group <45 years, IBD patients aged 45 to 65 years had lower odds of having a diagnosis of asthma (OR 0.68, 95% CI: 0.66-0.71). When compared to the Southern region, IBD patients with asthma in the Northeast region had higher odds of being hospitalized (OR: 1.54, 95% CI: 1.48-1.59). IBD patients with an ECI of 3 or more had higher odds of having a prior diagnosis of asthma compared to those with no comorbidities (OR 63.33, 95% CI: 54.51-73.58; Table 2). 

**Table 2:** Association between asthma and sociodemographic and clinical characteristics

Characteristics	OR (95% CI)	P value
Sex		
Female	1.00 [Reference]	
Male	0.58 (0.56-0.6)	<0.0001
Age (yr.)		
<45	1.00 [Reference]	
45-64	0.68 (0.66-0.71)	<0.0001
≥65	0.38 (0.36-0.39)	<0.0001
Race/ethnicity		
White	1.00 [Reference]	
Black	1.24 (1.19-1.29)	<0.0001
Hispanic	1.17 (1.11-1.25)	<0.0001
Other	1.02 (0.94-1.09)	0.69
Region		
South	1.00 [Reference]	
Northeast	1.54 (1.48-1.59)	<0.0001
Midwest	1.32 (1.27-1.36)	<0.0001
West	1.35 (1.30-1.40)	<0.0001
Elixhauser Comorbidity Index		
C0	1.00 [Reference]	
C1	18.25 (15.69-21.24)	<0.0001
C2	33.51 (28.82-38.97)	<0.0001
C3	63.33 (54.51-73.58)	<0.0001

Finally, having a prior diagnosis of asthma among hospitalized IBD patients was associated with lower odds of prolonged hospital stay and in-hospital mortality (OR 0.72, 95% CI: 0.69-0.74; OR 0.49, 95% CI: 0.43- 0.56, respectively; Table 3).

**Table 3:** Association between asthma and in-hospital mortality and prolonged hospitalization

Outcome variable	OR (95% CI)	P value
In-hospital mortality	0.49 (0.43-0.56)	<0.0001
Prolonged hospital stay	0.72 (0.69-0.74)	<0.0001

## Discussion

The prevalence of asthma in our hospitalized US IBD sample was 8%, which is similar to the general adult asthma population prevalence of 7.7% [3]. Patients with a higher ECI had higher odds of having a diagnosis of asthma as compared to those with no comorbidities. Multiple studies have highlighted the increased predisposition and higher susceptibility to other immune-mediated diseases in patients with one established autoimmune disease [4-5]. Hospitalized IBD patients with a history of asthma had a shorter LOS in hospital and lower in-hospital mortality as compared to IBD patients without an asthma diagnosis. Earlier and more aggressive treatment modalities administered to such patients with more comorbidities might explain such findings. In addition, since both IBD and asthma share autoimmune and inflammatory disease processes, some medications (e.g. corticosteroids) are effective for the control of both conditions. Patients with both IBD and asthma are more likely to seek medical care and have access to several providers, which may explain the lower in-hospital mortality in this group. The geographic difference in having a diagnosis of asthma among hospitalized IBD patients is in agreement with prior studies on asthma geographic variation [6]. The strength of this study is the large sample size used for power analysis of the association between IBD and asthma. Another strength is the focus on in-hospital mortality and prolonged hospital stay as main outcome variables and quality measures. The utilization of region in the multilevel regression also accounts for potential environmental factors that can account for the interplay between IBD and asthma. Limitations of this study include its retrospective nature and the difficulty to account for patients with multiple admissions as NIS focuses on unique hospitalizations rather than unique patients. In addition, only the association between the two diseases can be concluded, and no causation can be predicted since it is an observational model.

## Conclusions

Geographic variation and lower in-hospital mortality are notable in the outcomes of IBD patients with asthma, and the use of concomitant immunosuppressive medications may play a role in improved hospital outcomes. A focus on potential factors that drive improved outcomes should be sought. More prospective studies are needed to better understand the management and interplay of IBD patients with asthma. 
